# Naturally Occurring Radioactive Materials in Bracelets and Necklaces: Radiological Risk Evaluation

**DOI:** 10.3390/ijerph182111170

**Published:** 2021-10-24

**Authors:** Halmat Jalal Hassan, Suhairul Hashim, Noor Zati Hani Abu Hanifah, Sib Krishna Ghoshal, Mohamad Syazwan Mohd Sanusi, Fariza Hanim Binti Suhailin, Muhammad Fahmi Rizal Abdul Hadi, Rozman Mohd Tahar, David Andrew Bradley

**Affiliations:** 1 Department of Physics, Faculty of Science, Universiti Teknologi Malaysia, Skudai 81310, Johor, Malaysia; halmat.hassan@univsul.edu.iq (H.J.H.); nzatihani@gmail.com (N.Z.H.A.H.); sibkrishna@utm.my (S.K.G.); mohamadsyazwan@utm.my (M.S.M.S.); farizahanim@utm.my (F.H.B.S.); 2 Department of Physics, College of Education, University of Sulaimani, Sulaimani 46001, Kurdistan, Iraq; 3 Ibnu Sina Institute for Scientific and Industrial Research (ISISIR), Universiti Teknologi Malaysia, Skudai 81310, Johor, Malaysia; 4 School of Physics, Universiti Sains Malaysia, Gelugor 11800, Pulau Pinang, Malaysia; fahmi.riz04@gmail.com; 5 Atomic Energy Licensing Board, Jalan Dengkil, Batu 24, Dengkil 43000, Selangor, Malaysia; rozman@aelb.gov.my; 6 Centre for Applied Physics and Radiation Technologies, School of Engineering and Technology, Sunway University, Bandar Sunway 47500, Selangor, Malaysia; d.a.bradley@surrey.ac.uk; 7 Department of Physics, University of Surrey, Guilford GU2 7XH, UK

**Keywords:** NORMs, bracelets, necklaces, Geant4 MC, radiological risk

## Abstract

A particular category of jewelry is one involving bracelets and necklaces that are deliberately made to contain naturally occurring radioactive material (NORM)—purveyors making unsubstantiated claims for health benefits from the release of negative ions. Conversely, within the bounds of the linear no-threshold model, long-term use presents a radiological risk to wearers. Evaluation is conducted herein of the radiological risk arising from wearing these products and gamma-ray spectrometry is used to determine the radioactivity levels and annual effective dose of 15 commercially available bracelets (samples B1 to B15) and five necklaces (samples N16 to N20). Various use scenarios are considered; a Geant4 Monte Carlo (Geant4 MC) simulation is also performed to validate the experimental results. The dose conversion coefficient for external radiation and skin equivalent doses were also evaluated. Among the necklaces, sample N16 showed the greatest levels of radioactivity, at 246 ± 35, 1682 ± 118, and 221 ± 40 Bq, for ^238^U, ^232^Th, and ^40^K, respectively. For the bracelets, for ^238^U and ^232^Th, sample B15 displayed the greatest level of radioactivity, at 146 ± 21 and 980 ± 71 Bq, respectively. N16 offered the greatest percentage concentrations of U and Th, with means of 0.073 ± 0.0002% and 1.51 ± 0.0015%, respectively, giving rise to an estimated annual effective dose exposure of 1.22 mSv, substantially in excess of the ICRP recommended limit of 1 mSv/year.

## 1. Introduction

Ionizing radiation sources that exist naturally in the environment are dominated by the primordial terrestrial radionuclides ^232^Th and ^238^U, their progeny (forming the so-called decay series) and ^40^K. To be recognized is that in varying activity concentrations, these exist in all raw materials [1], sometimes being referred to as naturally occurring radioactive material (NORM). In association with extractive-, associated benefications-, and energy production industries, the activity levels of ^232^Th and ^238^U in naturally existing raw materials may be anthropogenically enhanced, in many circumstances typically appearing within the processing and utilization residues. In accord with the linear no-threshold model, doses exceeding local natural radiation background levels are linked with an increased radiological risk, a matter raising public health concerns [2,3]. Those same residues are sometimes being seen to be recycled, various external radiation sources, including naturally occurring radionuclides being deliberately included in consumer products, offered for various reasons, including not only matters of practical utility but also claimed increase in well-being [4]. Indeed, radioactive materials have for many years been incorporated in a range of consumer products, those proffered on the basis of a suggested enhancement of well-being forming the main focus of this work, particularly in regard to safety concerns [5]. Chronic exposure from frequent use of these radioactive consumer products (RCP) is both viewed to increase radiation risk to the public as well as potentially impact the environment as a result of discards [6]. Although the number of consumer products containing radioactive substances is currently relatively few in type, their production is seen to be ever-increasing [2].

Regarding RCP and the exemption dose limit for members of the public, in many countries, guidelines have been established, pointing to a need to satisfy the criteria of justification, optimization, and limitation, also authorization, not least in respect of NORM added consumer products [7]. The intent has typically been to introduce measures to avoid exposures exceeding the annual dose limit 1 mSv/year as recommended by the International Commission for Radiation Protection (ICRP) [8], a particular instance being the Malaysian Atomic Energy Licensing Board (AELB) technical document LEM/TEK/69 that specifically addresses consumer products containing sources of radioactivity [9]. Despite such measures, globally harmonized regulations have yet to be established in controlling the radioactive content of consumer products [10], many types of RCP being available for use in daily life, with products posing radiation risk from both external and internal exposure. Reports that exposure to natural radiation leads to beneficial health effects remains a contentious issue among researchers [11]. Here, one calls attention to products that purveyors are calling ionic bracelets and necklaces, items typically containing monazite and zircon at enhanced levels of radioactivity. The suggestion is that through the regular wearing of these items the associated chronic receipt of low dose-rate radiation gives rise to health benefits [12]. The manufacturers refer to this as negative ion technology [13]. Such claimed benefits include: improved circulation, stamina, and flexibility, the ability to detoxify and enhance energy levels, and a link to the prevention of cancer. In addition, such products claimed to contain germanium, have been suggested to produce far-infrared radiation (FIR). This they seek to link with studies that point to FIR therapy offering potential in aiding skin blood flow and in the reduction of heart diseases [14].

The radioactivity of mineral concentrates can be significantly influenced by small amounts of other minerals, such as monazite, containing elevated concentrations of ^232^Th and its progeny [15]. Monazite can contain up to 27% of U and Th oxides while zircon can contain U and Th oxides in concentrations up to 20% [16]. The IAEA technical document No. 1660 presents the range of typical activity concentrations of ^232^Th in monazite, at 40–600 Bq/g [17]. From this, it observed that existing minerals such as monazite and zircon added to consumer products could enhance the otherwise very low activity concentration of radionuclides in such items.

Based on the linear no-threshold model and stemming from the associated recommendations of the ICRP, no safe level is offered [18]. Accordingly, in regard to RCP utilization, at a minimum, it is necessary to evaluate the external radiation exposure to members of the public. In what is to follow, Geant4 Monte Carlo (Geant4 MC) simulation, the primary method for assessing the absorbed doses from external radiation, was used to obtain the dose rate conversion coefficient (mSv/h per Bq). The dose rate conversion coefficient was computed with respect to male and female reference phantoms. The results are expressed in terms of equivalent organ dose and external dose rate measurements. The individual annual effective dose from radiation exposure was estimated based on the duration of exposure.

## 2. Materials and Methods

### 2.1. Measurement of Activity of ^238^U, ^232^Th, and ^40^K in the Particular Bracelets and Necklaces

**Figure 1:**
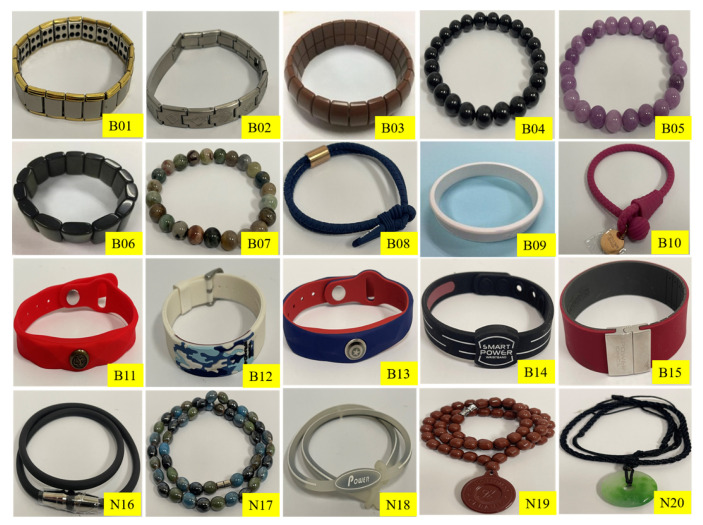
Bracelet and necklace samples.

A total of 15 bracelets and five necklaces were purchased online, noting manufacturer claims that the products generate negative ions, devoid of supporting scientific detail. Upon delivery, no evidence was found of information as to the radioactivity contained within. For analysis, these bracelets and necklaces were coded as B01-B15 and N16-N20, respectively (Figure 1). The activity of the individual radionuclides ^238^U, ^232^Th, and ^40^K contained within the samples were measured via gamma-ray spectrometry (using an ORTEC GEM Series P-type coaxial HPGe spectrometer, GEM20-76-LB-C-SMPCFG-SV-LB-76). The facility offers 33% relative efficiency and 1.8 keV full-width at half maximum (FWHM) at 1332 keV, providing spectroscopy within the photon energy range 40 keV to several MeV. Each sample was counted for a period of 24 h to ensure highly reliable counting statistics, and the measurement process was replicated twice for each sample. Gamma Vision 8.1 software was used for spectrum acquisition and analysis. Calibrations were made using a ^152^Eu standard point source, providing photon energies of 121.78, 244.6, 344.3, 411.1, 778.9, 867, 964, 1112, and 1528 keV. The activity of ^238^U was estimated from the primary sources ^214^Pb (295, 351) keV, and ^214^Bi (609, 1764) keV, as these line energies have a high probability emission of gamma [19]. Regarding ^226^Ra, the line energy 186 keV has not recorded significant difference activity from its daughters (^214^Pb and ^214^Bi) due to the secular equilibrium. Concerning the activity of ^232^Th, comprising ^228^Ra estimated from both gamma lines of ^228^Ac (338, 911) keV, ^228^Th estimated from the resulting gamma lines of ^212^Pb (238 keV) and ^212^Bi (727 keV); ^208^Tl (583, 2614) keV, with ^232^Th estimated from the average of ^228^Ra and ^228^Th.

### 2.2. Identification of Radioactive Materials Added in the Particular Bracelets and Necklaces

For analysis of elements contained in the purchased products, use was made of a Cartesian geometry energy dispersive X-ray fluorescence (ED-XRF) spectrometer (model; NEX CG–CG1240) [20,21,22]. High atomic number elements (Cs to U) are typically measured using L-line emissions, while lower atomic numbers element composition are generally analyzed using the K_α_ characteristic lines [23]. The system, with analytical software (RPF-SQX) featuring Rigaku Profile Fitting (RPF) technology, allows semi-quantitative analysis of most sample types without recourse to standards [13,24]. The samples were homogenized in powder form. ED-XRF analysis of seven such bracelets and necklaces was carried out for a duration of 12 min per sample (Table 1).

### 2.3. Estimation of the Annual Effective Dose from the Use of the Particular Bracelets and Necklaces

**Figure 2:**
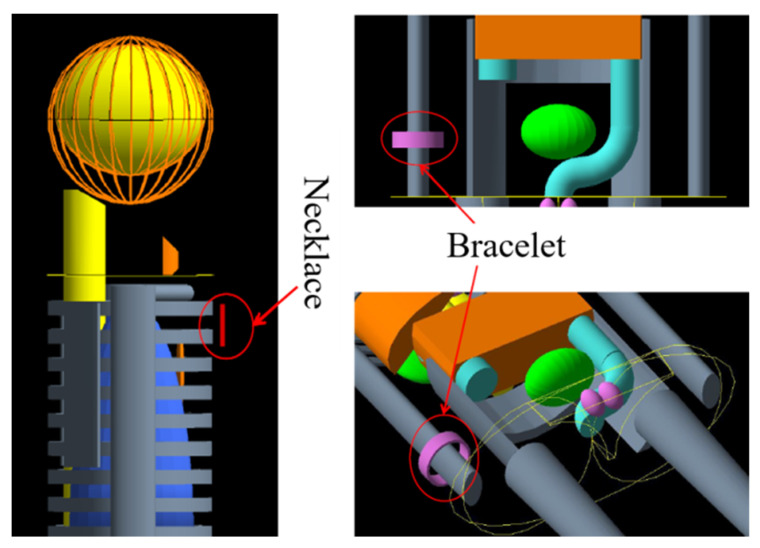
MIRD male human phantoms for AED bracelet and necklace estimates via Geant4 MC Simulation.

Clearly, several factors affect external radiation dose: source activity, shielding, distance, and period of use. To estimate the annual effective dose (AED) from the use of the bracelets and necklaces, simulation was conducted using the Geant4 Monte Carlo (Geant4 MC) radiation transport code version 10.06 patch 3, Physics list: Geant4 electromagnetic (EM) physics (G4EmStandardPhysics_option4), also involving the use of the Medical Internal Radiation Dose Pamphlet 5 (MIRD5) mathematical male and female adult phantoms, the male version being shown as an example in Figure 2 [25,26,27]. Direct gamma radiation from external exposures from ^232^Th and ^238^U series nuclides was simulated, also for the single emission of ^40^K (1460 keV). In consideration of radon dose, as has been noted, bracelets and necklaces are typically considered to be worn only during the day (the time periods over which gamma doses have been evaluated). During such periods of time, the wearer would not be anticipated to remain within a single room of small dimension and low ventilation. In such circumstances, any contributions to radon build from the bracelets would be considered negligible compared to ambient levels.

The bracelets were simulated at the position of the wrist while the necklaces were simulated at the position of the neck, conducted for both male and female phantoms [28].

Dose evaluations were carried out with the bracelets and necklaces located at a 1 mm distance from the covered skin surface. The annual effective dose for external exposure ($E_{ext}$) can be expressed as [12]:

(1) $$E_{ext}\;\left(mSv/y\right)=C_{n\;}\times D_{ext}\times E_{t}$$

where $E_{ext}\;\left(mSv/y\right)$ is the annual effective dose for external exposure, $C_{n\;}\;$is the activity of nuclide n (Bq), $D_{ext}$ is the external dose rate conversion factor (mSv/h per Bq) and $E_{t}$ is the annual exposure time (number of hours per year) as indicated in Table 2.

The skin equivalent dose rate $H_{skin}$ (μSv/h) due to use of the bracelets and necklaces was calculated using [2]:

(2) $$H_{skin}\left(\mu Sv/h\right)=\underset{n}{\sum }DCF_{skin}\times C_{n}\times W_{T}$$

where$\;DCF_{skin}$ is the skin dose conversion factor (μSv/h per Bq), at a distance of 1 mm, $C_{n}$ is the activity of nuclide n (Bq) and $W_{T}$ denotes the tissue weighting factor, with a value 0.01 for skin from ICRP-103; only gamma radiations were simulated, accordingly with a radiation weighting factor of unity (1) (Table 3). 

The recorded equivalent dose rates (in mSv/h) and radionuclides identified in the bracelets and necklaces are summarized in Table 4. The annual effective dose (AED) from the bracelets and necklaces was also evaluated based on the use of a calibrated portable detector (Identifinder 2, FLIR), obtained via [6]:

(3) AED (mSv/y)=Equivalent dose rate (mSv/h)×annual usage time (h/y)

## 3. Results and Discussion

In regard to the total activity within each of the bracelets and necklaces, Table 5 shows the results between sample values to be highly variable. For the bracelets (B01–B15), the greatest activity was found to be that in sample B15, at 146 ± 21 and 980 ± 71 Bq for ^238^U and ^232^Th, respectively. In contrast, sample B01 recorded very much lower activity, at respective values of 3.16 ± 0.7 and 3.5 ± 0.3 Bq. The range for ^40^K was between 10.5 ± 2 to 297 ± 55 Bq. In regard to the necklace samples (N16–N20), N16 (a rubber-based necklace) showed the greatest activity at 246 ± 35, 1682 ± 118, and 221 ± 40 Bq, for ^238^U, ^232^Th, and ^40^K, respectively. The gemstone necklace sample N17 recorded the next highest activity at 172 ± 24 and 1075 ± 89 Bq, for ^238^U and ^232^Th, respectively.

**Figure 3:**
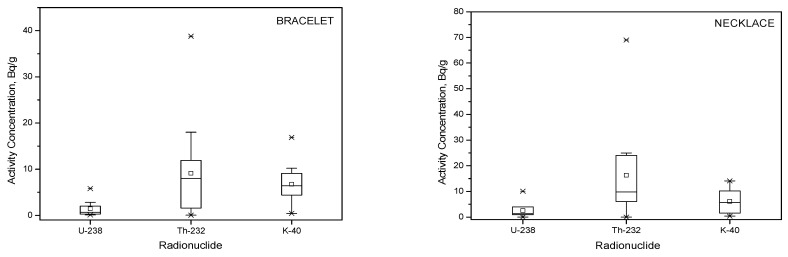
Overall distribution of activity concentration for ^238^U, ^232^Th, and ^40^K in bracelet and necklace samples.

Figure 3 shows a boxplot analysis of the activity concentration (Bq/g) of ^238^U, ^232^Th, and ^40^K in such bracelet and necklace samples, representing an overall distribution, incorporating data from Joseph et al. [12], Jang et al. [29], and Lee et al. [27], in addition to values from the current study. For the bracelet category, the lower-, median-, and upper-whisker are 0.09, 0.68, and 4.8 Bq/g for ^238^U, 0.04, 8, and 38 Bq/g for ^232^Th, and 0.42, 6.4, and 14.1 Bq/g for ^40^K, respectively. In respect of the necklace category, the results are 0.01, 1.3, and 4.8 Bq/g for ^238^U, 0.01, 9.83, and 69 Bq/g for ^232^Th, and 0.35, 5.7, and 14.1 for ^40^K, respectively. In some cases, the outliers are substantial, being greatest in respect of ^232^Th for both categories of jewelry; in the current study, the substantial outliers are due to the samples B15 and N16. For ^238^U, referring to the IAEA No. GSR Part 3 activity concentration of 10 Bq/g for exemption, current study results remain within that range [30].

Activity concentration of primordial radionuclides in bracelets and necklaces from literature data and present study findings are displayed in Table 6.

Table 1 lists the elemental content values of the bracelets and necklaces, varying significantly between products as expected. The concentrations for U and Th range from 21 ± 2 ppm through to 0.073 ± 0.0002% and from 60 ± 1 ppm through to 1.51 ± 0.0015%, respectively, while the % concentration of Zr was 1.33 ± 0.001. Via conversion (1 ppm is equal to 4.06 Bq/kg for Th and 12.35 Bq/kg for U) [31], the respective activity of U and Th for sample N16 was 218.5 ± 1.2 Bq, and 1487 ± 1.5 Bq, followed by the B15 wristband bracelet, the activity of U and Th being 109 ± 1 Bq and 743 ± 1 Bq, respectively. As displayed in Table 7, the total activity for U and Th, between the HPGe results of Table 5 and ED-XRF results from Table 1, are comparable.

The results reveal the bracelets and necklaces to contain elevated amounts of monazite and zircon, indicated by the content of the rare earths Ce, La, Nd, Sm, and Zr, these pointing to the source of radioactivity.

As apparent, sample N16 recorded a considerably greater level of radioactivity compared to other samples. The literature data for such bracelets and pendants Mubarak et al. [32] record a range of activity of 1-3189 and 1-884 Bq, for Th and U, respectively. Moreover, Furuta [2] in Japan recorded radioactivity in bath rock samples that contain monazite from China, at 1300 and 190 Bq/g for Th and U, respectively [2]. IAEA technical report 419 indicated evidence that zircon from China may contain significant elevations in the concentration of radioactivity, additionally with radionuclides concentration in monazite of up to 450 and 60 Bq/g for Th and U, respectively [15]. In the present study, the results reveal the high activity levels due to monazite and zircon contained in the samples. In regard to such products claiming to contain germanium, Table 1 shows no evidence of this element in the present range of bracelets and necklaces.

Table 2 presents the annual effective dose of ionic bracelets and necklaces for three different exposure durations (2 h, 8 h 7 min, and 16 h/day). The exposures for a period of exercise of 2 h/day, show sample N16 giving the highest annual effective dose at 1.53 × 10^−1^ mSv/year. The lowest annual effective dose was found to be that for metallic bracelet B01, at 1.36 μSv/year, giving rise to exposures less than the annual dose limit of 1 mSv/year for members of the public [7,30]. For a standard exposure time scenario, suggested to be 8 h 7 min/day [27], necklace N16 gives rise to the greatest annual effective dose of 6.21 × 10^−1^ mSv/year for adult male and female phantoms, remaining within the exemption limit. For a chronic exposure time scenario of say 16 h/day, pointing to the wearing of the products for the whole day but not including sleeping time, bracelet B15 and necklace N16 give rise to annual doses of 1.20 and 1.22 mSv/year, respectively. Accordingly, exceeding the public dose limit. From literature for necklaces, Joseph et al. [12] recorded 1.11 mSv/year for adults, while Jang et al. [29] estimated annual doses for bracelets and necklaces of 0.87 and 0.687 mSv/year respectively for an adult. Present MC simulation results have been compared with the work of Lee et al. [27], using the ICRP reference phantom and MCNPX.

Table 3 shows the dose rate and annual skin equivalent dose from wearing the present bracelets and necklaces, obtained using skin dose conversion coefficient. The highest skin equivalent dose rate $H_{skin}$ (μSv/h) has been observed for necklace N16, at 4.18 × 10^−1^ μSv/h, at a distance of 1 mm between the source and the covered surface skin. At a wearing time of 16 h per day, the highest annual skin equivalent dose was found to be for N16, at 2.44 mSv/year, while sample B15 recorded the next greatest value at 2.42 mSv/year. Overall the annual skin equivalent dose is less than the public limit of 50 mSv/year for skin [18] due to the small area of exposed skin.

Table 4 displays the dose rates and annual effective dose from bracelet and necklace products obtained using a calibrated IdentiFinder 2 portable detector (FLIR Survey Meter), recording equivalent dose rates in µSv/h and identifying the radionuclides contained within the bracelets and necklaces. For samples B15 and N16, these are found to give rise to an annual dose in excess of the dose limit of 1 mSv/year. The highest equivalent dose rate was 0.235 µSv/h for sample N16. The annual dose in use for 16 h/day achieves a value of 1.37 mSv/year for necklace N16, consistent with the Geant4 MC results.

In summary, the annual dose from the use of the bracelets and necklaces studied herein has been evaluated using the Geant4 MC codes, skin equivalent conversion coefficient, and identiFinder 2 survey meter, comparison being made for results for external dose. In regard to exemption from regulatory control, the IAEA safety standard series No. GSR Part 3 addresses the effective dose incurred by any individual in respect of an exempt practice or exempt source, at 10 μSv/year or less per product [7]. The European Commission 147 guidelines similarly proposes an effective dose for users of consumer products arising from normal use, again not exceeding 10 μSv/year per product [7,33]. Also noted is that the use of radioactive materials in consumer products has been regulated by the European Union [34], not allowing radioactive materials to be added to personal jewelery. Based on the European Commission guidelines, with the exception of sample B01, all of the bracelets and necklaces herein exceed the 10 μSv/year exemption limit. In respect of the ICRP guidance limit of 1 mSv/year annual dose for members of the public, several samples herein exceed that limit in their use of 16 h/day and more. As reported in ICRP-103, unjustified exposures can be applied to a range of consumer products NORM-added. If the benefits of using a consumer product NORM-added cannot be shown to exceed the risk, then a ban would seem to be required [2,34], as would certainly seem to be the case for the presently investigated products. A strong recommendation is made to prohibit radioactive consumer products that exceed the exemption limit.

## 4. Conclusions

This paper reports on the radioactivity in so-called ionic bracelets and necklaces, measured using an HPGe detector. The activity values were then used to simulate doses in male and female phantoms, computing the dose conversion coefficient, and then performing external radiation dose assessment. Various use scenarios were adopted in evaluating the annual dose. Samples B15 and N16 recorded the greatest level of radioactivity, ^238^U, ^232^Th, and ^40^K being is to be greatest in sample N16. Effective dose evaluation and skin equivalent conversion coefficient results were obtained for external exposure using the MIRD5 phantom. It has been concluded that close contact and chronic use of these consumer products can infer annual effective doses of up to 1.22 mSv/year, exceeding the dose constraint of 1 mSv/year for members of the public. While an absence of any justification for using these bracelets and necklaces is apparent, the online purchase of these products is still available in some countries, including Malaysia. Due to the elevated levels of radioactivity found in these products and insufficient available data on any health benefit, recommendation is made to ban such products from import and sale.

## Figures and Tables

**Table 1:** Elemental concentrations of the bracelets and necklaces. (Quoted in ppm for the purpose of consistency).

Sample	Elemental Concentration ± Standard Deviation (ppm)								
	Zr	Ce	La	Nd	Sm	U	Th	K	Ge
B03	1940 ± 8	435 ± 31	ND	ND	ND	149 ± 2	2200 ± 5	49,400 ± 51	ND
B06	440 ± 5	331 ± 16	ND	ND	ND	21 ± 2	60 ± 1	61,700 ± 38	ND
B09	13,300 ± 10	ND	ND	ND	ND	ND	1110 ± 2	1580 ± 23	ND
B13	2440 ± 8	306 ± 15	167 ± 16	35 ± 11	192 ± 16	94 ± 1	3650 ± 7	291 ± 15	ND
B15	4500 ± 3	125 ± 7	40 ± 5	ND	229 ± 20	350 ± 1	7250 ± 10	357 ± 23	ND
N16	6900 ± 13	445 ± 32	419 ± 24	ND	480 ± 23	729 ± 2	15,100 ± 15	737 ± 28	ND
N17	7850 ± 2	554 ± 3	ND	1310 ± 57	981 ± 39	272 ± 1	4320 ± 4	6560 ± 19	ND

ND: Not Detected.

**Table 2:** Annual effective dose from present bracelets and necklaces for three different exposure times.

Sample	Annual Effective Dose (mSv/Year)		
	Exercise (2 h/Day)	Standard (8 h 7 min/Day)	Chronic (16 h/Day)
B01	1.36 × 10^−3^	5.51 × 10^−3^	1.09 × 10^−2^
B02	8.30 × 10^−3^	3.36 × 10^−2^	6.64 × 10^−2^
B03	8.90 × 10^−2^	3.61 × 10^−1^	7.12 × 10^−1^
B04	1.11 × 10^−2^	4.5 × 10^−2^	8.88 × 10^−2^
B05	1.22 × 10^−2^	4.95 × 10^−2^	9.76 × 10^−2^
B06	1.97 × 10^−2^	7.99 × 10^−2^	1.57 × 10^−1^
B07	1.1 × 10^−2^	4.46 × 10^−2^	8.8 × 10^−2^
B08	2.26 × 10^−2^	9.17 × 10^−2^	1.81 × 10^−1^
B09	2.55 × 10^−2^	1.03 × 10^−1^	2.04 × 10^−1^
B10	1.30 × 10^−2^	5.27 × 10^−2^	1.04 × 10^−1^
B11	1.50 × 10^−2^	6.08 × 10^−2^	1.20 × 10^−1^
B12	1.80 × 10^−2^	7.31 × 10^−2^	1.44 × 10^−1^
B13	5.12 × 10^−2^	2.08 × 10^−1^	4.1 × 10^−1^
B14	5.80 × 10^−2^	2.35 × 10^−1^	4.64 × 10^−1^
B15	1.50 × 10^−1^	6.09 × 10^−1^	1.2
N16	1.53 × 10^−1^	6.21 × 10^−1^	1.22
N17	9.64 × 10^−2^	3.91 × 10^−1^	7.72 × 10^−1^
N18	1.49 × 10^−2^	6.05 × 10^−2^	1.19 × 10^−1^
N19	2.83 × 10^−2^	1.15 × 10^−1^	2.26 × 10^−1^
N20	8.85 × 10^−3^	3.59 × 10^−2^	7.08 × 10^−2^

**Table 3:** Dose rate and annual skin equivalent dose of bracelets and necklaces obtained using the skin dose conversion coefficient.

Sample	Dose Rate (μSv/h)	Annual Skin Equivalent Dose (mSv/Year)		
		2 h/day	8 h 7 min/Day	16 h/Day
B03	2.21 × 10^−1^	1.61 × 10^−1^	6.53 × 10^−1^	1.29
B06	4.15 × 10^−2^	3.03 × 10^−2^	1.23 × 10^−1^	2.42 × 10^−1^
B09	6.23 × 10^−2^	4.55 × 10^−2^	1.85 × 10^−1^	3.64 × 10^−1^
B13	1.30 × 10^−1^	9.50 × 10^−2^	3.86 × 10^−1^	7.60 × 10^−1^
B15	4.15 × 10^−1^	3.03 × 10^−1^	1.23	2.42
N16	4.18 × 10^−1^	3.05 × 10^−1^	1.24	2.44
N17	8.48 × 10^−2^	6.19 × 10^−2^	2.51 × 10^−1^	4.95 × 10^−1^

**Table 4:** Dose rates and annual effective dose from bracelet and necklace products, obtained using the IdentiFinder 2 portable detector.

Sample	Dose Rate (µSv/h)	Annual Dose (mSv/Year)			
		2 h/day	8 h 7 min/Day	16 h/Day	Radionuclides
B15	0.213	0.155	0.631	1.24	Th/U
N16	0.235	0.172	0.696	1.37	Th/U

**Table 5:** Total activities (in Bq) for ^238^U, ^232^Th, and ^40^K for each of the bracelets and necklaces.

Sample	Description	Weight ± SD (g)	Activity ± SD* (Bq)		
			^238^U	^232^Th	^40^K
B01	Metallic bracelet	25.3 ± 0.25	3.16 ± 0.7	3.5 ± 0.3	10.5 ± 2
B02	Metallic quantum bracelet	35.3 ± 0.3	3.2 ± 0.6	23.4 ± 2	72 ± 13
B03	Gemstone bracelet (brown)	41.7 ± 0.4	106 ± 14	428 ± 35	297 ± 55
B04	Gemstone bracelet (black)	20 ± 0.19	4.9 ± 0.6	32 ± 4.3	93 ± 15
B05	Gemstone bracelet (pink)	18.92 ± 0.18	5.4 ± 0.4	41 ± 5.5	92 ± 13
B06	Gemstone bracelet (green)	51.6 ± 0.4	15.8 ± 2.6	18.3 ± 1.7	253 ± 45
B07	Multi gemstone bracelet	19 ± 0.18	3.9 ± 0.8	29 ± 4.3	83.6 ± 15
B08	Rob bracelet (blue)	11.49 ± 0.1	14 ± 2.3	98 ± 18	100 ± 18
B09	Rubber bracelet (white)	12.96 ± 0.13	16 ± 3	115 ± 9	105 ± 18
B10	Rob bracelet (pink)	5.59 ± 0.05	4 ± 1	46 ± 4	93 ± 16
B11	Rubber bracelet (red)	16.83 ± 0.17	11 ± 1	56 ± 3	94 ± 16
B12	Rubber bracelet (white)	11.7 ± 0.1	12 ± 1	73 ± 3	106 ± 16
B13	Rubber bracelet (blue)	16.27 ± 0.16	24 ± 2.3	290 ± 21	109 ± 19
B14	Rubber bracelet (black)	11.16 ± 0.1	53 ± 7	319 ± 24	113 ± 20
B15	Wristband bracelet smart power (dark red)	25.21 ± 0.2	146 ± 21	980 ± 71	83 ± 15
N16	Rubber necklace (black)	24.26 ± 0.2	246 ± 35	1682 ± 118	221 ± 40
N17	Multi gemstone necklace	42.94 ± 0.3	172 ± 24	1075 ± 89	95 ± 17
N18	Rubber necklace (grey)	10.57 ± 0.1	10 ± 1.6	110 ± 7.3	97 ± 17
N19	Quantum necklace (brown)	53.62 ± 0.5	43 ± 3.2	281 ± 14	84 ± 6.5
N20	Gemstone necklace (green)	7.05 ± 0.07	9 ± 0.5	43 ± 7	73 ± 13

SD*: Standard deviation.

**Table 6:** Activity of radionuclides ^238^U, ^232^Th, and ^40^K in bracelets and necklaces. Literature data and data from this study.

No	Sample	Activity Concentration ± SD (Bq/g)			References
		^238^U	^232^Th	^40^K	
1	Bracelet	4	20	2	[12]
2	Bracelet	NA	15.1	14.1	[27]
3	Bracelet	0.1–2.81	0.04–11.9	NA	[29]
4	Bracelet	0.01 ± 0.01–5.8 ± 0.8	0.14 ± 0.01–38 ± 3	0.4 ± 0.1–16.9 ± 3	Present study
5	Necklace	4	24	NA	[12]
6	Necklace	NA	15	14	[27]
7	Necklace	0.01–1.6	0.01 -10	0.4–2	[29]
8	Necklace	0.8 ± 0.05–10 ± 1.5	5.4 ± 0.3–69 ± 5	2 ± 0.4–10.4 ± 1.8	Present study

NA: Not Available.

**Table 7:** Total activity ± SD (in Bq) for U and Th. A comparison between the results of Table 5 (HPGe) and Table 1 (ED-XRF).

Sample	HPGe		ED-XRF	
	U	Th	U	Th
B03	106 ± 14	428 ± 35	76.73 ± 1	372.5 ± 0.8
B06	15.8 ± 2.6	18.3 ± 1.7	13.4 ± 1.3	12.5 ± 0.2
B09	16 ± 3	115 ± 9	ND	58 ± 0.4
B13	24 ± 2.3	290 ± 21	18.9 ± 0.4	241 ± 0.5
B15	146 ± 21	980 ± 71	109 ± 1	743 ± 1
N16	246 ± 35	1682 ± 118	218.5 ± 1.2	1487 ± 1.5
N17	172 ± 24	1075 ± 89	144 ± 1	754 ± 0.7
